# GSK-3β/β-TrCP regulates Nrf2-mediated oxidative stress response in *Cristaria plicata*

**DOI:** 10.1016/j.isci.2025.113995

**Published:** 2025-11-11

**Authors:** Yile Hu, Jinhua An, Han Qiu, Qinglian Wu, Jianqing Li, Gang Yang, Baoqing Hu, Chungen Wen

**Affiliations:** 1 College of Life Science, Key Laboratory for Aquatic Germplasm Innovation and Utilization of Jiangxi Province, Education Ministry Key Laboratory of Poyang Lake Environment and Resource Utilization, Nanchang University, Nanchang 330031, China

**Keywords:** Biochemistry, Molecular biology

## Abstract

Nuclear factor erythroid 2-related factor 2 (Nrf2) orchestrates antioxidant and cytoprotective responses. Beyond classical Keap1-dependent redox regulation, Nrf2 activation can occur via the GSK-3β/β-TrCP pathway during Keap1 inhibition. This study investigated this non-canonical pathway in the stress adaptation of the bivalve (*Cristaria plicata*). We found that the *Cp*GSK-3β and *Cp*β-TrCP genes underwent significant changes in the hepatopancreas, gills, and kidneys after immune (LPS, PGN, Poly(I:C)) or oxidative (H_2_O_2_) challenges. Knockdown of either gene increased Nrf2 and NQO1 expression. Protein interaction assays confirmed *Cp*GSK-3β/*Cp*β-TrCP binding to the Neh6 domain of *Cp*Nrf2, with *Cp*GSK-3β phosphorylating this domain *in vitro*. Overexpression enhanced cellular resistance to H_2_O_2_. These findings reveal the conserved physiological significance of the GSK-3β/β-TrCP-Nrf2 axis in molluscan stress responses, providing new insights into redox regulation mechanisms in invertebrates and potential biomarkers for aquatic environmental stress assessment.

## Highlights

• First cloning of the GSK-3β and β-TrCP genes in *Cristaria plicata*
• The protein structure and function of GSK-3β and β-TrCP in *Cristaria plicata* were confirmed
• To explore the role of GSK-3β and β-TrCP in Nrf2

## Graphical abstract

**Figure:**
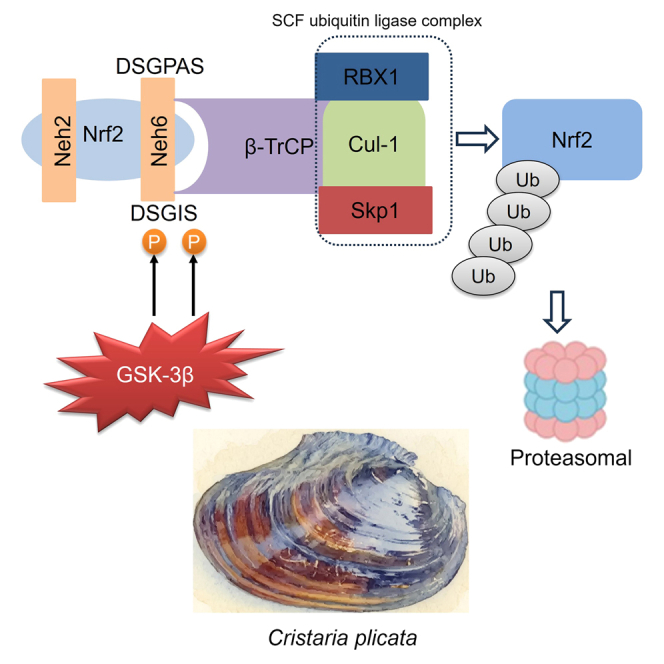

## Introduction

Nrf2 plays an integral role in maintaining redox homeostasis, which also controls cell growth, and is degraded by at least two ubiquitin-proteasome pathways. One is through a redox-dependent manner, under normal redox homeostasis, the ETGE and DLG motifs in Neh2 domain of Nrf2 interact with Keap1 and then bind to Cul3Rbx1/Roc1 to ubiquitinate Nrf2.^1^^,^^2^ While the organisms expose to the following stressors such as tumors in liver, stomach, lungs, etc, Keap1 inhibition by thiol-active compounds, and the ubiquitination of Nrf2 mediated by Keap1 disappear.^3^^,^^4^ Another approach to regulate Nrf2 is identified through a redox-independent manner. The C-terminal DSGIS and N-terminal DSGPAS of Nrf2 Neh6 domain is recognized by F box after WD40 substrate adaptor of β-TrCP. Subsequent ubiquitination of Nrf2 by SCF (Skp1-cullin-1-F-box) β-TrCP. Different from Neh2 domain require for ubiquitination of CRL Keap1, and the function of two motifs Neh6 domain are independent.^4^

GSK-3β is one of the isoforms of glycogen synthase kinase 3, and is a highly conserved serine/threonine kinase that not only regulates glycogen synthesis, but also participates in cellular processes, such as inflammation, immune function, and convergence of multiple signaling pathways.^5^^,^^6^ The activity of GSK-3β is regulated by substrate-specific mechanisms, including protein complex formation, substrate phosphorylation, phosphorylation, and subcellular localization.^7^ GSK3β regulates Nrf2-mediated anti-oxidative stress in two ways. One way is a direct way to directly phosphorylate Nrf2 Neh6 structure and serine 335 and 338 sites in cytoplasm to promote Nrf2 into nucleus, and is recognized by E3 ligase β-TrCP leads to Nrf2 ubiquitination and degradation. Another way is an indirect way, the activated GSK3β can phosphorylate the threonine of Fyn and enter into nucleus. After Fyn is activated, Nrf2 tyrosine 568 site is phosphorylated, and leads to Nrf2 nuclear export and degradation.^8^^,^^9^

Beta-transducin repeat-containing protein (β-TrCP) is an important component of SCF-type ubiquitin ligase, and plays a key role in the ubiquitination process.^10^ β-TrCP is mainly involved in cell cycle progression, metabolism, immunity and inflammation, and contains an F box domain that interacts with Skp1. Seven WD40 repeat motifs that are E3 substrate binding regions, and form into a propeller.^11^^,^^12^ The substrates of β-TrCP are divided into two categories, cell cycle regulators and pro-apoptotic regulators, which participate in various life processes in cells by mediating the ubiquitination and degradation of different substrates.^13^^,^^14^^,^^15^ Most classical substrates contain consensus sequence DpSGX1-3pS (X is any amino acid) or its variants. β-TrCP recognizes substrates, and the subsequent ubiquitination requires specific kinases to phosphorylate serine residues. The atypical substrate contains the consensus sequence DDGXXD of the aspartic acid residue that replaces phosphorylated serine, and β-TrCP does not need to be phosphorylated in advance for recognition.^16^^,^^17^^,^^18^

GSK-3β promotes proteasome degradation and activates NF-κB through phosphorylation targets, thereby resisting apoptosis in cytoplasm, which phosphorylates DNA repair factors and promotes DNA repair in nucleus,^19^ and interferes with oxidative stress-mediated apoptosis by releasing cytochrome *c* from mitochondria through calpain I.^20^ In the Wnt signaling pathway, β-TrCP can recognize the phosphorylated DSGXXS motif in β-catenin and degrade it by ubiquitination, thereby reducing the expression level and nuclear accumulation of β-catenin and effectively inhibiting the transcriptional activity of downstream target genes.^21^ The effect of GSK-3β/β-TrCP pathway on Nrf2 is verified in GSK-3 knockout mice and the DSGIS sequence in Nrf1 can be degraded by β-TrCP in nucleus.^3^ Curcumin combined with HUC-MSC treatment can polarize microglia into an anti-inflammatory phenotype through AKT/GSK-3β/β-TrCP/Nrf2 signaling pathway, thereby promoting the neurological function recovery of AIS. While phosphorylation occurs at the Ser473 site of AKT that leads to phosphorylation at the Ser9 (inactive state) site of GSK3β, inhibits the ubiquitination of β-TrCP, and leads to Nrf2 output from nucleus to cytoplasm.^22^ Ca^2^^+^ dependent GSK3β activation ubiquitinates Nrf2 through β-TrCP-Cul1 E3 ligase complex.^23^ GSK-3β is identified from *Epinephelus coioides*, which can promote the replication of SGIV in GS cells and negatively regulates interferon-related factors.^24^ The domains of GSK-3β1 and 2 in *Ctenopharyngodon idella* indicate that they may have the same function as mammals in teleost fish and have different effects on lipid accumulation in adipose tissue and liver.^25^ β-TrCP regulates the stability of β-catenin in *Xenopus laevis* and plays a role in its embryonic development.^26^

In aquatic invertebrates, including mollusks, the Nrf2/Keap1 pathway is also a crucial regulator of antioxidant responses. For instance, in bivalves such as mussels and clams, Nrf2 homologs are involved in counteracting oxidative stress induced by environmental pollutants and bacterial challenges.^27^^,^^28^ Studies have shown that oxidative stress plays a central role in the immune response of bivalves against bacterial infections, and the activation of Nrf2-mediated pathways is essential for enhancing their antioxidant capacity during such stresses.^29^ In mollusks, glycogen synthase kinase-3 beta (GSK-3β) serves as a critical and versatile signaling node, integrating inputs from diverse pathways to regulate both immune response and metabolic processes.^30^^,^^31^ Its function is highly dependent on cellular context and the initiating stimulus. In the immune realm, as demonstrated in the oyster Crassostrea gigas, GSK-3β (CgGSK3β) is a key substrate in an innate immune pathway. Upon bacterial recognition by the C-type lectin receptor CgCLec-TM1, the MAPK ERK (CgERK) is activated and subsequently phosphorylates CgGSK3β at its Ser9 residue. This phosphorylation event is an essential step that ultimately induces the production of pro-inflammatory cytokines CgIL-17-1 and CgIL-17-5 to combat infection.^31^ Conversely, in the context of metabolism, as shown in the pearl oyster Pinctada fucata, GSK-3β is a downstream target of the insulin signaling pathway.^32^ Activation of the insulin receptor by hrIGF-I stimulates the PI3K/Akt pathway, which leads to the phosphorylation and inhibition of GSK-3β. This inhibition results in the decreased mRNA expression of GSK-3β itself and the phosphorylation of glycogen synthase, a direct target of GSK-3β. This coordinated action decreases glucose levels in hemocytes and increases glycogen storage in digestive glands, highlighting its central role in regulating glycogen metabolism.^33^ Thus, GSK-3β in mollusks acts as a pivotal regulatory hub, transducing signals from both immune and metabolic receptors to orchestrate appropriate physiological responses, from cytokine production to energy storage.

*C. plicata* widely distributes in large rivers, lakes and reservoirs, which is an important economic species in aquaculture production in China, is also cultivated in freshwater pearl industry, and is called a filter feeder in aquatic ecosystems.^34^^,^^35^^,^^36^ Due to overexploitation and the presence of sewage, the health of bivalves is seriously threatened. However, little is known about GSK-3β/β-TrCP-Nrf2 pathway in molluscs. In this study, we determined and analyzed the full-length sequences of GSK-3β and β-TrCP genes from *C. plicata* (designated as *Cp*GSK-3β and *Cp*β-TrCP). The expression levels of *Cp*GSK-3β and *Cp*β-TrCP in different tissues and under the stimulation of bacteria and hydrogen peroxide were analyzed by qPCR. The expression of *Cp*Nrf2 and *Cp*NQO1 was detected after knocking down *Cp*GSK-3β and *Cp*β-TrCP genes. The binding of *Cp*Nrf2-Neh6 to *Cp*GSK-3β and *Cp*β-TrCP proteins was detected by yeast two-hybrid and GST-pulldown. The phosphorylation of *Cp*Nrf2-Neh6 by *Cp*GSK-3β was detected *in vitro*. The resistance of *Cp*GSK-3β and *Cp*β-TrCP to H_2_O_2_ was proved by disk diffusion and cell viability experiments. These results may provide a theoretical basis for studying the role of GSK-3β/β-TrCP-Nrf2 pathway in molluscs immune stress.

## Results

### CDNA and sequence analysis of *Cp*GSK-3β and *Cp*β-TrCP

The full length of *Cp*GSK-3β was 2152 bp, 5′ and 3′ UTR was 370 and 519 bp, respectively. ORF was 1263 bp and encoded 420 amino acids with a predicted molecular weight of 46.5 kDa (Figure S1A). *Cp*GSK-3β had the highest identity of 99% with Hyriopsis cumingii (Figure S1C). Multiple sequence alignment showed that the vertebrates and invertebrates of *Cp*GSK-3β are divided into one branch, and Hydra vulgaris is a branch. Among them, *Cp*GSK-3β *C. plicata* and *H. cumingii* are clustered into one branch. (Figure S2A).

The full length of *Cp*β-TrCP was 2998 bp, 5′ and 3′ UTR was 92 and 515 bp, respectively. ORF was 2391 bp and encoded 796 amino acids with a molecular weight of 89.6 kDa (Figure S1B). *Cp*β-TrCP protein had the highest identity of 67% with Crassostrea gigas (Figure S1D). Homologous multiple sequence alignment showed that *Cp*β-TrCP was closely related to β-TrCP of Mercenaria mercenaria (Figure S2B).

### Tissue expression of *Cp*GSK-3β and *Cp*β-TrCP

**Figure 1:**
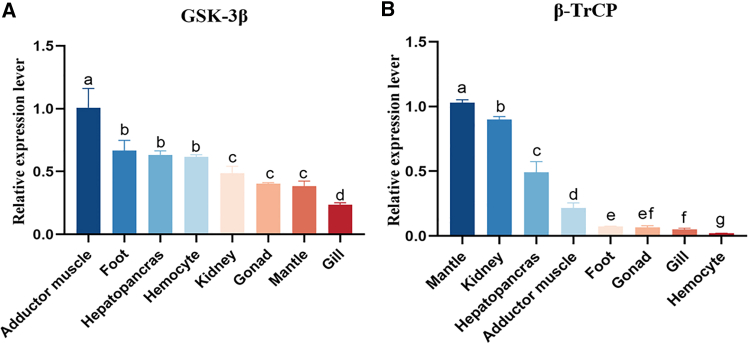
Tissue-specific expression of GSK-3β and β-TrCP in *C. plicata* Gene expression analysis of *Cp*GSK-3β and *Cp*β-TrCP across different tissues of the *C. plicata*. (A) Relative expression levels of *Cp*GSK-3β. (B) Relative expression levels of *Cp*β-TrCP. Within each panel, bars sharing the same lowercase letter are not significantly different, whereas bars marked with different letters indicate statistically significant differences (Student’s *t*-test, *p* < 0.05). Data are presented as the mean ± SD from three independent biological replicates (*n* = 3).

The expression of *Cp*GSK-3β was relatively high in hepatopancreas, adductor muscle, foot, and hemocyte, and the lowest expression was in gill (Figure 1A). The expression of *Cp*β-TrCP was higher in hepatopancreas, mantle and kidney, and the lowest expression was in hemocyte (Figure 1B).

### The expression of *Cp*GSK-3β and *Cp*β-TrCP mRNA after bacterial stimulation

**Figure 2:**
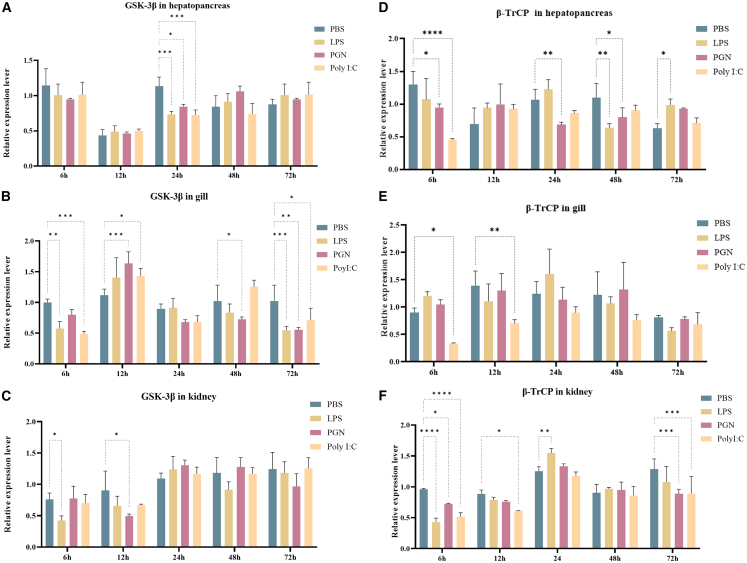
Expression changes of *Cp*GSK-3β and *Cp*β-TrCP following immune stimulation (A) Changes in *Cp*GSK-3β in the hepatopancreas following stimulation of mussels with LPS, PGN, or Poly(I:C). (B) Changes in *Cp*GSK-3β expression in the gills at different time points following LPS, PGN, or Poly(I:C) stimulation. (C) Changes in *Cp*GSK-3β in the kidney. (D) Changes in *Cp*β-TrCP in the hepatopancreas following stimulation of mussels with LPS, PGN, or Poly(I:C). (E) Changes in *Cp*β-TrCP expression in the gills at different time points following LPS, PGN, or Poly(I:C) stimulation. (F) Changes in *Cp*β-TrCP in the kidney of *C. plicata.* Data are presented as mean ± SD (*n* = 3). Statistical significance was determined by *t*-tests and is denoted as follows: ∗*p* ≤ 0.05, ∗∗*p* ≤ 0.01, ∗∗∗*p* ≤ 0.001, ∗∗∗∗*p* ≤ 0.0001.

Following stimulation with LPS, PGN, and Poly(I:C), both GSK-3β and β-TrCP genes in *C. plicata* exhibited a trend toward downregulation. Compared to the PBS group, GSK-3β showed significant changes after stimulation with LPS, PGN, and Poly(I:C), displaying an overall downregulated trend. In the hepatopancreas, *Cp*GSK-3β was significantly downregulated at 12 h (Figure 2A). In the gills, *Cp*GSK-3β was markedly downregulated at 6, 24, and 48 h (Figure 2B). In the kidneys, *Cp*GSK-3β was significantly downregulated primarily at 6 and 12 h (Figure 2C). Similarly, following stimulation with LPS, PGN, and Poly(I:C), β-TrCP mRNA in *C. plicata* also exhibited a significant downregulation trend. Specifically, in the hepatopancreas, *Cp*β-TrCP was significantly downregulated at 6, 24, and 48 h (Figure 2D). In the gills, *Cp*β-TrCP was significantly downregulated at 6 and 12 h following Poly(I:C) stimulation (Figure 2E). In the kidneys, *Cp*β-TrCP was significantly downregulated at 6, 12, and 72 h (Figure 2F). In summary, *Cp*GSK-3β and *Cp*β-TrCP participate in immune responses triggered by LPS, PGN, and Poly(I:C), playing a crucial role in innate immunity.

### The expression of *Cp*GSK-3β and *Cp*β-TrCP mRNA after hydrogen peroxide stimulation

**Figure 3:**
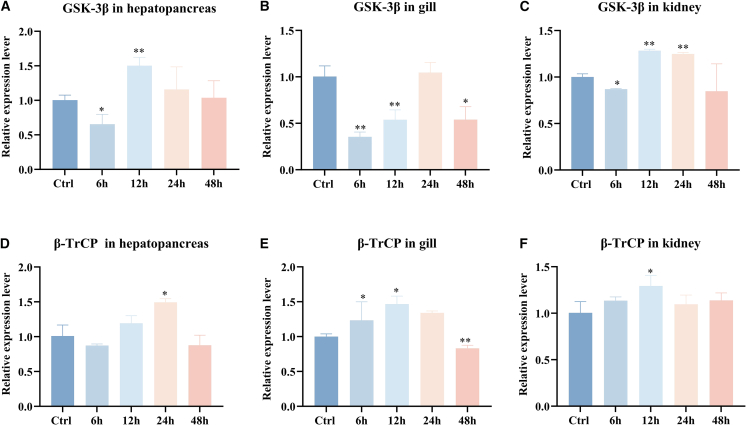
Expression of *Cp*GSK-3β and *Cp*β-TrCP in *C.plicata* following hydrogen peroxide stimulation (A) Expression levels of *Cp*GSK-3β in the hepatopancreas of mussels after hydrogen peroxide stimulation. (B) Expression levels of *Cp*GSK-3β in the gills of mussels after hydrogen peroxide stimulation. (C) Expression levels of *Cp*GSK-3β in the kidneys of mussels after hydrogen peroxide stimulation. (D) Expression levels of *Cp*β-TrCP in the hepatopancreas of mussels after hydrogen peroxide stimulation. (E) Expression levels of *Cp*β-TrCP in the gills of mussels after hydrogen peroxide stimulation. (F) Expression levels of *Cp*β-TrCP in the kidney of mussels after hydrogen peroxide stimulation. Data are presented as mean ± SD (*n* = 3). Statistical significance was determined by *t*-tests and is denoted as follows: ∗*p* ≤ 0.05, ∗∗*p* ≤ 0.01.

Following H_2_O_2_ exposure, *Cp*GSK-3β exhibited a biphasic pattern—initial downregulation followed by recovery (Figures 3A–3C). Its transcript levels dropped markedly at 6 h in the hepatopancreas, gill, and kidney of Cristaria plicata, then rebounded by 24 h. Conversely, *Cp*β-TrCP displayed the opposite trend: a transient upregulation succeeded by a decline (Figures 3E–3F). At 12–24 h, *Cp*β-TrCP expression was significantly elevated in all three tissues relative to the control, but by 48 h it had uniformly decreased.

### The effect of interfering *Cp*GSK-3β and *Cp*β-TrCP on downstream genes

**Figure 4:**
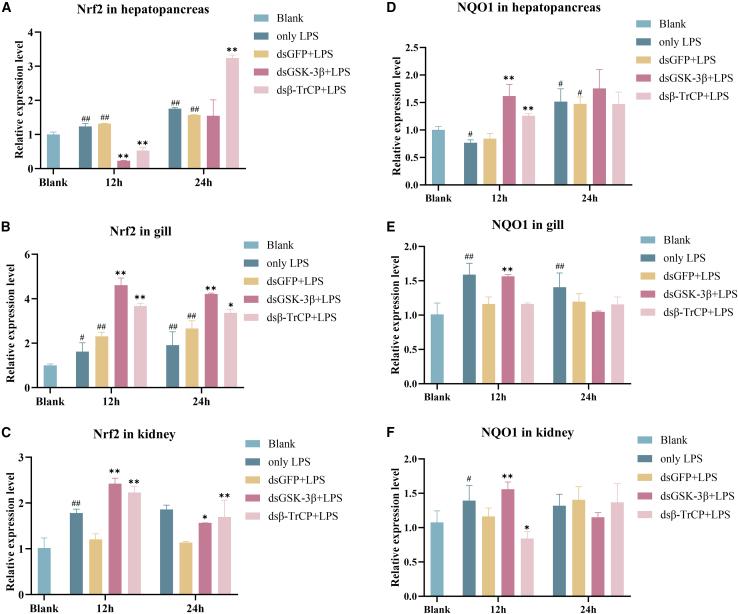
Effects of *Cp*GSK-3β and *Cp*β-TrCP knockdown on *Cp*Nrf2 and *Cp*NQO1 expression in different tissues (A) The gene of *Cp*Nrf2 expression in hepatopancreas following ds*Cp*GSK-3β. (B) The gene of *Cp*Nrf2 expression in gills following ds*Cp*GSK-3β. (C) The gene of *Cp*Nrf2 expression in kidney following ds*Cp*GSK-3β. (D) The gene of *Cp*NQO1 expression in hepatopancreas following ds*Cp*GSK-3β. (E) The gene of *Cp*NQO1 expression in gills following ds*Cp*GSK-3β. (F) The gene of *Cp*NQO1 expression in kidney following ds*Cp*GSK-3β.

Under non-stimulated conditions, compared to the control group, RNA interference targeting GSK-3β and β-TrCP resulted in significantly reduced expression in the hepatopancreas, gills, and kidneys, with significant downregulation observed at both 12 and 24 h (Figures S3A–S3F). Under LPS stimulation, compared to the blank group, LPS stimulation elevated GSK-3β expression, with more pronounced changes observed in the hepatopancreas and kidneys. Compared to the LPS+ ds*Cp*GFP (negative control) group, the LPS+ ds*Cp*GSK-3β group demonstrated the highest interference efficiency for GSK-3β in the hepatopancreas, with reductions of 68.7% and 38.5% at 12 h and 24 h, respectively (Figures S3G–S3I). β-TrCP also exhibited a similar downward trend under LPS stimulation. The most pronounced downregulation occurred in the hepatopancreas and gills at 24 h, decreasing by 62.56% and 68.95%, respectively (Figures S3J–S3L). After injection, the expression of related genes in hepatopancreas, gill and kidney was detected at 12 and 24 h. The results showed that *Cp*Nrf2 was significantly up-regulated in hepatopancreas and kidney after injection of ds*Cp*GSK-3β and ds*Cp*β-TrCP, which significantly down-regulated after injection of ds*Cp*β-TrCP in gill, and significantly decreased after injection of ds*Cp*GSK-3β at 24 h (Figure 4A). *Cp*NQO1 increased in all three tissues after injection of ds*Cp*GSK-3β and ds*Cp*β-TrCP (Figure 4B).

### Localization of *Cp*GSK-3β and *Cp*β-TrCP in cells

**Figure 5:**
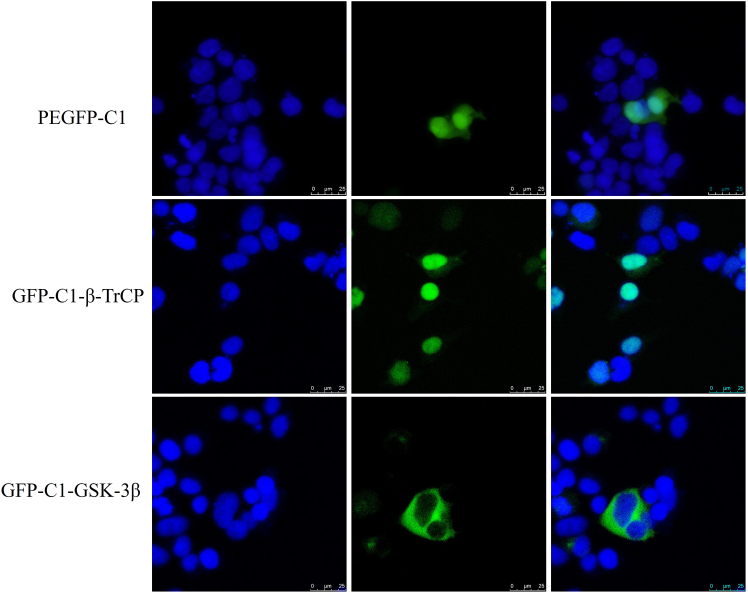
Localization of *Cp*GSK-3β and *Cp*β-TrCP in HEK-293T cells Scale bar: 25 μm.

The fusion proteins of pEGFP-C1-*Cp*GSK-3β and pEGFP-C1-*Cp*β-TrCP were transfected into HEK-293T cells to determine the localization of *Cp*GSK-3β in cells. The results showed that *Cp*GSK-3β was mainly localized in cytoplasm, and *Cp*β-TrCP was distributed in nucleus and cytoplasm (Figure 5).

### Expression and purification of recombinant protein

**Figure 6:**
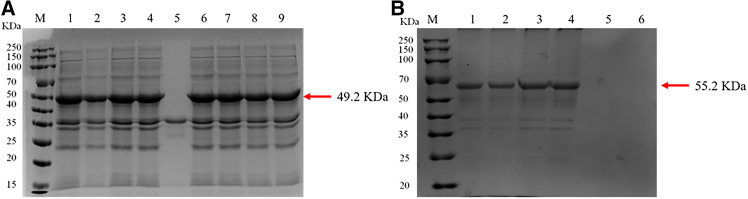
SDS-PAGE analysis of protein purification (A) SDS-PAGE analysis of *Cp*GSK-3β protein purification. (B) SDS-PAGE analysis of *Cp*β-TrCP protein purification.

The results showed that the significant difference did not have in the elution effect of *Cp*GSK-3β recombinant protein with 500 mM-1 M imidazole concentration (Figure 6A). Its molecular weight was consistent with the predicted value of 49.2 kDa (with His tag). The recombinant protein of *Cp*β-TrCP (Figure 6B) was eluted with an imidazole concentration of 500 mM–800 mM, which was consistent with the predicted protein molecular weight. Since the active site of this protein is primarily located within its domain region, only the domain portion was constructed as a recombinant protein. Its molecular weight matched the predicted value of 55.2 kDa (with SUMO tag).The recombinant proteins of *Cp*GSK-3β and *Cp*β-TrCP with concentrations of 0.531 g/L and 0.495 g/L were obtained.

### Yeast two-hybrid verification

**Figure 7:**
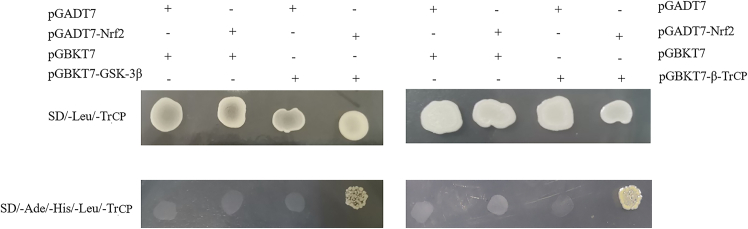
The binding of *Cp*Nrf2 to *Cp*GSK-3β and *Cp*β-TrCP was detected by yeast two-hybrid

The screened colonies (AD + BK, AD-Nrf2 + BK, AD + BK-GSK-3β, AD + BK-β-TrCP, AD-Nrf2 + BK-GSK-3β, AD-Nrf2 + BK-β-TrCP). The cells were inoculated on 2-deficient (SD/-Leu/-Trp) and 4-deficient (SD/-Leu/-Trp/-His/-Ade) plates and were cultured at 30°C for 2–3 days. The results showed that only AD-Nrf2 + BK-GSK-3β and AD-Nrf2 + BK-β-TrCP colonies grew normally on 4-deficient plates, indicating that Nrf2 binds to GSK-3β and β-TrCP, respectively (Figure 7).

### Interaction of recombinant proteins

**Figure 8:**
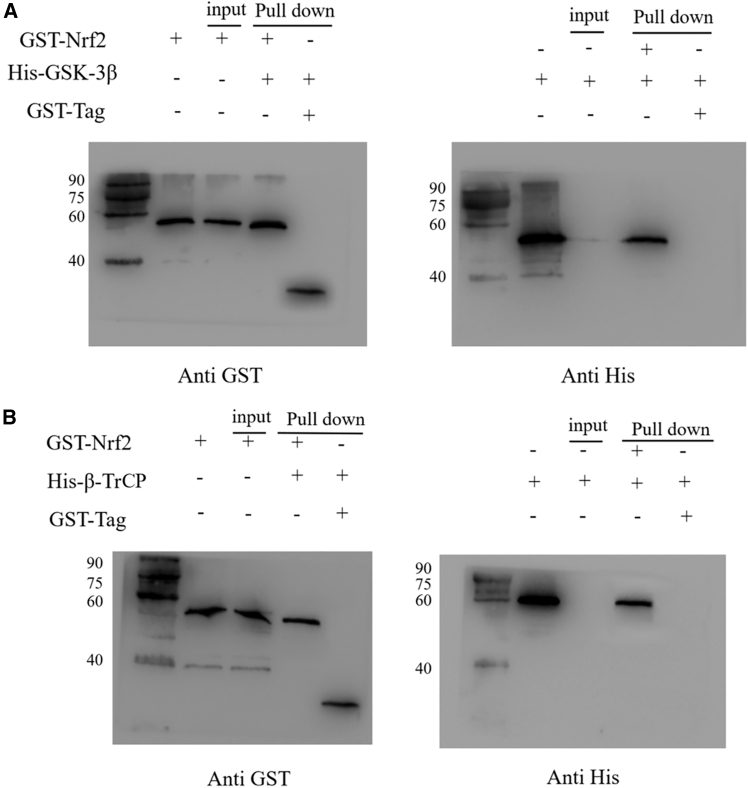
Protein-protein interactions were validated using the GST pull-down assay (A) Interaction between *Cp*Nrf2 and *Cp*GSK-3β. (B) Interaction between *Cp*Nrf2 and *Cp*β-TrCP.

GST-*Cp*Nrf2 protein and GST-Tag protein were detected in the group of GST-*Cp*Nrf2 + His-*Cp*SK-3β, GST-*Cp*Nrf2 + His-*Cp*β-TrCP and GST-Tag + His-*Cp*GSK-3β, GST-Tag + His-*Cp*β-TrCP co-incubation with anti-GST tag mouse monoclonal antibody. The His-*Cp*Nrf2 + His-*Cp*SK-3β and His-*Cp*β-TrCP fusion proteins were detected in GST-*Cp*Nrf2 + His-*Cp*SK-3β and GST-*Cp*Nrf2 + His-*Cp*β-TrCP co-incubation groups by anti-His-tagged mouse monoclonal antibody, while no His-*Cp*GSK-3β and His-*Cp*β-TrCP fusion proteins were detected in GST-Tag + His-*Cp*GSK-3β and GST-Tag + His-*Cp*β-TrCP co-incubation groups. The results showed that there was no binding between GST-Tag and His-*Cp*GSK-3β and His-*Cp*β-TrCP, indicating that GST-*Cp*Nrf2 binds to His-*Cp*GSK-3β (Figure 8A) and His-*Cp*β-TrCP, respectively (Figure 8B).

### *Cp*GSK-3β phosphorylates *Cp*Nrf2-Neh6

**Figure 9:**
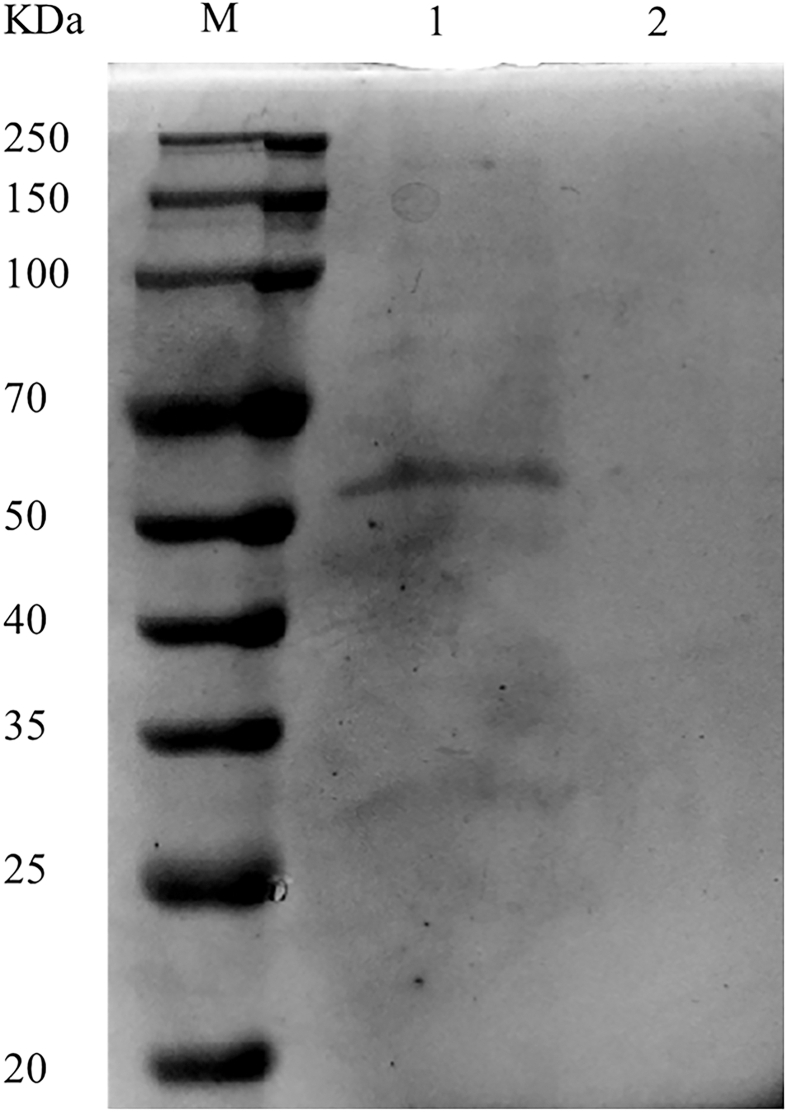
Detected the effect of *Cp*GSK-3β on the phosphorylation of *Cp*Nrf2-Neh6 *in vitro* Note: Lane 1 reaction solution contains *Cp*GSK-3β fusion protein, only *Cp*Nrf2-Neh6 protein was detected in Lane 2 reaction solution.

The results of SDS-PAGE and staining showed that the lane containing *Cp*GSK-3β protein had bands and only the lane containing *Cp*Nrf2-Neh6 protein had no bands. The results indicated that *Cp*GSK-3β fusion protein had phosphorylation effect on *Cp*Nrf2-Neh6 (Figure 9).

### Antioxidant activity of *Cp*GSK-3β and *Cp*β-TrCP

**Figure 10:**
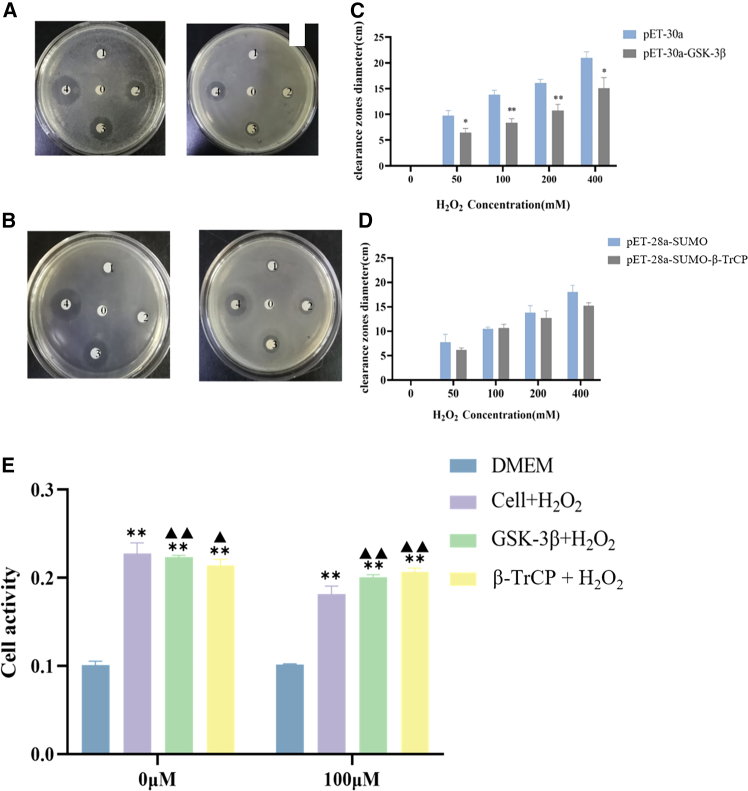
Expression of *Cp*GSK-3β and *Cp*β-TrCP under H_2_O_2_stress and its effect on *E. coli* BL21 cell viability (A–D) Expression at different H_2_O_2_ concentrations: 0 (ddH_2_O), 50 mM, 100 mM, 200 mM, and 400 mM. (E) Cell viability after H_2_O_2_ stimulation. Asterisks (∗) denote significant differences compared to the medium-only control group. Triangles (▲) indicate significant differences between the compared plasmid-transfected groups. Data are shown as mean ± SD (*n* = 3). P-values were determined by *t*-test: ∗/▲, *p* ≤ 0.05; ∗∗/▲.

In the absence of hydrogen peroxide, *E.coli* grew normally. In the presence of hydrogen peroxide, the growth inhibition zone increased with the increase of its concentration. While *Cp*GSK-3β (Figures 10A and 10C) and *Cp*β-TrCP (Figures 10B and 10D) recombinant proteins were transferred, the inhibition zone of E.coli was significantly reduced by comparison with the control group (pET-30a, pET-28a-SUMO).

After incubation with hydrogen peroxide for 6 h, CKK-8 reagent was added into the incubator at 37°C for half an hour and then was detected at 450 nm using a microplate reader. The results showed that the survival rate of cells without plasmid was only 63.3% after H_2_O_2_ stimulation. While the survival rate of cells with pCMV-HA-*Cp*GSK-3β and pCMV-HA-*Cp*β-TrCP was increased by 80.9% and 92.9%, respectively (Figure 10E).

## Discussion

The molecular weight of GSK-3β in *E. coioides* is 48.23 KDa.^24^ Two GSK-3β homologous sequences are obtained in *C. idella*, and the molecular weights of GSK-3β1 and GSK-3β2 are 46.87 KDa and 50.18 KDa, respectively.^25^ In humans, the molecular sizes of β-TrCP1 and β-TrCP2 are 69 KDa and 62 kDa, respectively.^37^ The predicted molecular weights of *Cp*GSK-3β and *Cp*β-TrCP were 46.6 KDa and 89.6 KDa, respectively. In vertebrates, GSK-3 has two subtypes, GSK3α and GSK3β, both of which have serine/threonine kinase domains, but the difference is that the N-terminus of GSK3α is composed of 63 glycine-rich residues.^38^ In this study, *Cp*GSK3β and vertebrate GSK3β was the same internal node by phylogenetic tree analysis. The GSK3β of *H. cumingii* and *Cp*GSK3β were clustered into a branch, indicating that *Cp*GSK3β belonged to the β-isomer of GSK3 family. In addition, multiple sequence comparisons showed that the S_TKc domain of *Cp*GSK-3β had high homology with other species, indicating that *Cp*GSK-3β might have similar regulatory effects with mammalian GSK-3β.

The highly conserved β-TrCP from *Drosophila melanogaster* to humans is encoded by a single gene in invertebrates, while two subtypes of β-TrCP1 and β-TrCP2 are in mammalian genome.^39^^,^^40^ The N-terminus of mammalian β-TrCP has an F box motif and the C-terminus has seven WD40 repeats.^14^ The F box connects β-TrCP and other SCF complex components, while WD40 recruits the substrate to SCF.^39^ The results of multiple sequence alignment showed that the structure of *Cp*β-TrCP was the same as that of mammals. So, it was speculated that *Cp*β-TrCP had a similar role to the mammalian domain. Research on GSK-3β and β-TrCP in invertebrates remains limited. Structurally, the catalytic domain of GSK-3β exhibits >90% amino acid homology across Drosophila, *Caenorhabditis elegans*, and humans, with its key phosphorylation sites (Ser9/Tyr216) and substrate recognition sequence being fully conserved.^41^ The F box and WD40 repeat domains of β-TrCP exhibit nearly identical structures across invertebrates and vertebrates, enabling identical recognition of DSG(X)NS disruption motifs.^12^ In terms of gene number, invertebrates are known to retain only one GSK-3 gene and one β-TrCP gene. Vertebrates acquired two homologs, GSK3α/β and β-TrCP1/2, following a whole-genome duplication event.^39^^,^^40^ Furthermore, the absence of the N-terminal 76-amino acid extension in invertebrate homologs explains why small-molecule inhibitors (LiCl, SB216763, and CHIR99021) exhibit slightly lower IC_50_ values in insect cell expression systems while maintaining similar selectivity profiles.^42^ In Drosophila embryo polarity establishment and sea urchin endoderm differentiation models, the GSK-3/SHAGGY-Slimb complex mediates β-catenin phosphorylation and degradation in a mechanism identical to that found in mammals59. These findings confirm the existence of a conserved “GSK-3β–β-TrCP–Nrf2” axis in invertebrates. In our study, the freshwater mussel Cristaria plicata possesses only a single GSK-3β homolog and lacks additional isoforms such as GSK-3α. Cristaria plicata GSK-3β and β-TrCP jointly modulate Nrf2 phosphorylation, thereby exerting antioxidant and anti-apoptotic effects.

In humans, the tissue expression of GSK-3β mRNA and protein is inconsistent. For example, GSK-3β mRNA is mainly expressed in testis, while protein is mainly expressed in lung and kidney.^38^ In addition, GSK-3β mRNA is expressed in all tissues of *E. coioides* and is abundantly expressed in skin, blood and intestine.^24^ The two homologous genes in *C. idella* are expressed in spleen, muscle, liver, adipose tissue, intestine, and heart, but GSK-3β1 is the highest in adipose tissue and GSK-3β2 is the highest in liver.^25^ *Cp*GSK-3β was expressed in all tissues, and *Cp*GSK-3β was highly expressed in adductor muscle, foot, hepatopancreas, and hemocyte. Cell division cycle 20 homolog (cdc20) containing WD40 domain was expressed in kidney and spleen of *Megalobrama amblycephala*.^43^ Similarly, smu-1 containing similar domains is expressed in gills and muscles of *Macrobrachium rosenbergii*. Fluorescence quantitative detection showed that *Cp*β-TrCP was expressed in all tissues of *C.plicata*, with the highest expression was in mantle and kidney, the lowest was in hemocyte. It meant that *Cp*GSK-3β and Cpβ-TrCP might be involved in regulating various biological functions of *C. plicata*.

The expression of GSK-3β in *C. idella* is upregulated at 6 h after injection of Poly(I:C), which return to normal level at 72 h and reached the highest level in liver at 6 h. After stimulation of CIK cells with Poly(I:C), the expression level also reach the highest level at 6 h.^44^ LPS stimulation promotes the binding of β-TrCP to PKD1, resulting in the downregulation of PKD1 and the recovery of IκBα protein level. These prove that β-TrCP may be a negative regulator of the upstream signal of the TLRs-NF-κB pathway.^45^ The non-classical degron motif of TIPE2 interacts with β-TrCP through TAK1 under LPS stimulation, followed by ubiquitination and degradation. The amount of TIPE2 in macrophages determines the degree of LPS-dependent signal transduction and gene expression, and the degradation of TIPE2 can prevent persistent inflammatory response.^46^ GSK-3β is activated by high glucose, the level of β-TrCP is higher in nucleus of diabetic rats, and the increased expression of β-TrCP in renal cells treated with high glucose is confirmed by immunocytochemistry.^47^ Bergenin can inhibit high glucose-induced β-TrCP expression.^48^ Hydrogen peroxide stimulation leads to GSK-3β activation and phosphorylation of Nrf2.^49^ In this study, we found that after stimulation with LPS, PGN, and Poly(I:C), the expression levels of *Cp*GSK-3β and *Cp*β-TrCP in the hepatopancreas, kidneys and gills were significantly downregulated. After hydrogen peroxide stimulation, the mRNA expression levels of *Cp*GSK-3β and *Cp*β-TrCP also changed significantly. The relative expression level of GSK-3β showed a downward trend at 6 h and an upward trend at 12 h. Mechanistically speaking, this might be related to the stimulating dose of hydrogen peroxide and the exposure time. In the early stage of hydrogen peroxide stimulation, the PI3K/Akt pathway is activated, resulting in an increase in phosphorylation at the Ser9 inhibitory site of GSK-3β and a decrease in GSK-3β activity.^50^ This may promote the stability of proteins such as Nrf2, β-catenin and CREB, inducing cells to enter an antioxidant and anti-apoptotic state. However, as the stimulation time increases, the cells gradually enter the pro-apoptotic/necrotic program. GSK-3β is activated, accelerating the degradation of Nrf2, inhibiting β-catenin, promoting the phosphorylation of Bax and p53, leading to uncontrolled ROS production and cell death.^51^ In conclusion, *Cp*GSK-3β and *Cp*β-TrCP play certain roles in oxidative stress and inflammatory responses.

Knockdown of β-TrCP1 and β-TrCP2 in Keap-mouse MEFs increase β-catenin and Nrf2 levels.^52^ The protein level of Nrf2 is increased after inhibiting GSK-3 and β-TrCP1 and 2 by siRNA.^3^ While GSK-3β is knocked down or GSK-3β inhibitor is used, Nrf2 is accumulated in nucleus, and the expression levels of antioxidant proteins are upregulated, including NQO1, HO1, and GCLC.^53^^,^^54^ In this study, the expression of Nrf2 and NQO1 was upregulated after injection of ds*Cp*GSK-3β and ds*Cp*β-TrCP, indicating that *Cp*GSK-3β and *Cp*β-TrCP were negatively correlated with Nrf2 and NQO1. It was indicated that GSK-3β/β-TrCP acted on Nrf2 through negative regulation.

Mammalian GSK-3β is located in cytoplasm, and GSK-3β of the Orange-spotted grouper is also located in cytoplasm.^24^^,^^55^^,^^56^ Subcellular localization results displayed that *Cp*GSK-3β was mainly localized in cytoplasm of HEK293T cells. It was indicated that *Cp*GSK-3β mainly regulated Nrf2 in cytoplasm. Two β-TrCP proteins exist in both cytoplasm and nucleus in human cells.^39^^,^^40^ In addition, the substrates of β-TrCP1 are distributed throughout the cytoplasm or nucleus, so the subcellular localization of β-TrCP1 is crucial for the selection of targets. Therefore, it must be finely regulated and GSK-3β plays a mediating role in the degradation of various proteins induced by β-TrCP.^57^ This study exhibited that *Cp*β-TrCP was distributed in nucleus and cytoplasm. It was suggested *Cp*β-TrCP played a role in both nucleus and cytoplasm.

The interaction between GSK3β and Nrf2 is found in cytoplasm of Huh7.5.1 cells by double fluorescence immunocytochemistry staining.^58^ Mammalian two-hybrid experiments and *in vitro* biotinylated peptides show that Nrf2-Neh6 interacted with β-TrCP1-WD40, and non-phosphorylated DSGIS and DSAPGS motifs can also be recognized by β-TrCP.^59^ Under the condition of keeping GSK-3β activity in cells, the co-transfection of Nrf2 and the chimera containing Neh6 structure combines with β-TrCP, which prove that the Neh6 domain of Nrf2 interacts with GSK-3/β-TrCP axis.^60^ In this study, yeast two-hybrid experiments showed that *Cp*Nrf2-Neh6 interacted with *Cp*GSK-3β and *Cp*β-TrCP, respectively. The results were further verified by GST-pulldown, and were the same as the former. GSK-3β can phosphorylate Nrf2-Neh6 both *in vivo* and *in vitro*.^47^^,^^61^^,^^62^ The bands appeared in the reaction solution containing *Cp*GSK-3β and *Cp*Nrf2-Neh6 protein. This indicated that *Cp*GSK-3β could be phosphorylated by *Cp*Nrf2-Neh6 *in vitro*. Currently, numerous studies have directly or indirectly demonstrated that GSK-3β can directly phosphorylate the serine residues of Nrf2, generating a phosphorylation-degradation signal.^4^^,^^59^ This signal is subsequently recognized by β-TrCP, leading to phosphorylation at the Tyr568 site of Nrf2.^59^ This process results in Nrf2 nuclear export and degradation. However, the serine residues of Nrf2 are located within the Neh6 domain.^49^ In our study, we demonstrated that GSK-3β in the crested river mussel can phosphorylate the Neh6 domain of *Cp*Nrf2. This finding is consistent with the aforementioned mechanism.

Research indicates that GSK-3β and β-TrCP have antioxidant functions.^4^ GSK-3β, a serine/threonine protein kinase, phosphorylates the Neh6 domain of Nrf2, which contains the DSGIS motif, enabling its recognition by β-TrCP.^59^ This phosphorylation facilitates Nrf2 ubiquitination and subsequent proteasomal degradation via the Cullin1/Rbx1 complex. β-TrCP acts as an adaptor for E3 ubiquitin ligases, recognizing GSK-3β-phosphorylated Nrf2 and promoting its ubiquitination and proteasomal degradation. Thus, both GSK-3β and β-TrCP directly regulate Nrf2 activity.^60^ After HEK293T cells are treated with 100 μM H_2_O_2_ for 2 h, the activity of GSK-3β is enhanced.^63^ The activities of β-TrCP1 and a small amount of β-TrCP2 in HEK293T cells are increased after H_2_O_2_ stimulation and co-immunoprecipitation with RCAN1-1 is observed.^64^ In this study, the expression of *Cp*GSK-3β and *Cp*β-TrCP proteins was employed for paper diffusion experiments. The results showed that both of them were resistant to H_2_O_2_ and the cells stimulated by H_2_O_2_ had higher cell survival rate after transfection with *Cp*GSK-3β-HA and *Cp*β-TrCP-HA. This indicated that *Cp*GSK-3β and *Cp*β-TrCP were involved in the antioxidant process of *C. plicata*. These were speculated that *Cp*GSK-3β/*Cp*β-TrCP resisted oxidative stress through Nrf2-Neh6. Therefore, we speculate that the increased cell survival rate following overexpression of *Cp*GSK-3β/*Cp*β-TrCP may be mediated through its regulation of the Nrf2 pathway.

In conclusion, we cloned GSK-3β and β-TrCP from *Cristaria plicata* and investigated the regulatory mechanisms through which these genes modulate Nrf2 under stress conditions. Both *Cp*GSK-3β and *Cp*β-TrCP were found to respond to immune challenges induced by LPS, PGN, Poly(I:C), and H_2_O_2_. Knockdown of *Cp*GSK-3β or *Cp*β-TrCP led to upregulation of Nrf2 and its downstream gene NQO1. Subcellular localization analysis in HEK293T cells showed that *Cp*GSK-3β is primarily localized in the cytoplasm, whereas *Cp*β-TrCP is distributed in both the cytoplasm and nucleus. Protein-protein interactions between *Cp*Nrf2-Neh6 and *Cp*GSK-3β or *Cp*β-TrCP were confirmed by yeast two-hybrid and GST pull-down assays. Furthermore, *Cp*GSK-3β was demonstrated to phosphorylate *Cp*Nrf2-Neh6 *in vitro*. Overexpression of *Cp*GSK-3β or *Cp*β-TrCP enhanced cell viability under H_2_O_2_-induced oxidative stress, indicating that both genes contribute to antioxidant defense mechanisms.

### Limitations of the study

As a lower invertebrate, the crested clam faces certain technical limitations in research. For instance, in gene knockdown validation experiments, the lack of species-specific antibodies makes it difficult to conduct effective validation at the protein level. Furthermore, no stable cell lines have yet been established for the crested clam, and related mechanism studies are still primarily conducted in 293T cells, indicating significant room for improvement in this research system.

## Resource availability

### Lead contact

Further information and requests for resources and reagents should be directed to and will be fulfilled by the lead contact, Chungen Wen (cgwen@ncu.edu.cn).

### Materials availability

All materials are available upon reasonable request.

### Data and code availability

• Data: Protein sequences required for phylogenetic tree construction and amino acid sequence alignment were obtained from the National Center for Biotechnology Information (NCBI) database. Their accession numbers are listed in the key resources table (Deposited data section).
• Code: This article does not report original code.
• Others: Any additional information required to reanalyze the data reported in this article is available from the lead contact upon request.

## Acknowledgments

This research was financially supported by grants (no. 31472305, 31460697, and 31660734) from 10.13039/501100001809National Natural Science Foundation of China, the support project of the 10.13039/501100015335Modern Agricultural Industry Technology System (ZQT20180027), Jiangxi Province Graduate Innovation Special Fund Project (YC2022-B023).

## Author contributions

Conceptualization, Y.H. and J.A.; methodology, H.Q., Q.W., and J.L.; investigation, Y.H. and, J.A.; writing—original draft, Y.H; writing—review and editing, C.W. and J.A.; funding acquisition, G.Y. and C.W.; resources, B.H. and G.Y.; supervision, B.H., C.W., and G.Y.

## Declaration of interests

The authors declare no competing interests.

## STAR★Methods

### Key resources table

**Table:** 

REAGENT or RESOURCE	SOURCE	IDENTIFIER
**Antibodies**		

6∗His, His-Tag Monoclonal antibody	proteintech	RRID: AB_1565792
ChromoTek rat anti GST Monoclonal antibody (6G9)	proteintech	RRID: AB_2455075

**Chemicals, peptides, and recombinant proteins**		

Trizol (RNAiso Plus)	TaKaRa	9109
HiScript II Q RT SuperMix for qPCR (+ gDNA wiper) kit	Vazyme	R223-01
LPS (Lipopolysaccharide)	Macklin	L861706-5mg
PGN	Macklin	P909273-10mg
Poly(I:C) (Polyinosinic-polycytidylic acid)	yuanye Bio-Technology	CAS#24939-03-5
H_2_O_2_ (Hydrogen peroxide)	Nanjing Reagent	C0404510123
T7 RNAi Transcription Kit	Vazyme	TR102
ChamQ Universal SYBR qPCR Master Mix	Vazyme	Q711-02
jetOPTIMUS® *in vitro* DNA transfection reagent	SARTORIUS	101000006
DAPI	Beyotime	C1002
LB Broth	CHINOOK	CN230276-250g
GST-Beads	Sangon Biotech	D510271-0500
PVDF	Millipore	IPVH00010
CCK-8 kit	Beyotime	C0038
Protein Stains S (phosphorylated protein gel detection kit)	Sangon Biotech	C500043-0010

**Deposited data**		

*Cp*GSK-3β	This paper	N/A
*Cp*β-TrCP	This paper	N/A
*Rattus norvegicus* (GSK-3β)	NCBI database	NP_114469.1
*Xenopus laevis* (GSK-3β)	NCBI database	NP_001083752.1
*Bos taurus* (GSK-3β)	NCBI database	NP_001094780.1
*Homo sapiens* (GSK-3β)	NCBI database	NP_002084.2
*Sus scrofa* (GSK-3β)	NCBI database	NP_001121915.1
*Danio rerio* (GSK-3β)	NCBI database	NP_571456.1
*Hydra vulgaris* (GSK-3β)	NCBI database	AEM76871.1
*Crassostrea gigas* (GSK-3β)	NCBI database	XP_011452760.1
*Crassostrea angulata* (GSK-3β)	NCBI database	CCN27373.1
*Mizuhopecten yessoensis* (GSK-3β)	NCBI database	OWF50471.1
*Monopterus albus* (GSK-3β)	NCBI database	XP_020465114.1
*Octopus sinensis* (GSK-3β)	NCBI database	XP_029636743.1
*Gallus gallus* (GSK-3β)	NCBI database	XP_040516343.1
*Nibea albiflora* (GSK-3β)	NCBI database	KAG8000603.1
*Hyriopsis cumingii* (GSK-3β)	NCBI database	UYI35619.1
*Mercenaria mercenaria* (GSK-3β)	NCBI database	XP_045207912.1
*Daphnia magna* (β-TrCP)	NCBI database	JAM74715.1
*Homo sapiens* (β-TrCP)	NCBI database	KAI4077194.1
*Mus musculus* (β-TrCP)	NCBI database	AAD41025.1
*Xenopus tropicalis* (β-TrCP)	NCBI database	NP_001016386.1
*Bos taurus* (β-TrCP)	NCBI database	DAA14816.1
*Equus asinus* (β-TrCP)	NCBI database	XP_014693125.1
*Octopus sinensis* (β-TrCP)	NCBI database	XP_029644679.1
*Pomacea canaliculate* (β-TrCP)	NCBI database	XP_025093821.1
*Mizuhopecten yessoensis* (β-TrCP)	NCBI database	OWF43849.1
*Crassostrea gigas* (β-TrCP)	NCBI database	XP_011415998.2
*Crassostrea virginica* (β-TrCP)	NCBI database	XP_022341985.1
*Aplysia californica* (β-TrCP)	NCBI database	XP_012941965.2
*Alligator sinensis* (β-TrCP)	NCBI database	XP_025051050.1
*Danio rerio* (β-TrCP)	NCBI database	XP_005173126.1
*Mercenaria mercenaria* (β-TrCP)	NCBI database	XP_045164454.1
*Ostrea edulis* (β-TrCP)	NCBI database	XP_048740230.1

**Experimental models: Cell lines**		

293T	Procell system	CL-0005

**Experimental models: Organisms/strains**		

*Cristaria plicata*	Education Ministry Key Laboratory of Poyang Lake Environment and Resource Utilization	Poyang Lake

**Oligonucleotides**		

See Table S1	This paper	N/A

**Recombinant DNA**		

GEP-C1-β-TrCP	This paper	N/A
GFP-C1-GSK-3β	This paper	N/A
pGADT7-Nrf2	This paper	N/A
рGBKT7-GSK-3β	This paper	N/A
рGBKT7-β-TrCP	This paper	N/A

**Software and algorithms**		

Expasy	SIB Swiss Institute of Bioinformatics	https://web.expasy.org/translate/
BLAST	NCBI database	https://blast.ncbi.nlm.nih.gov/BLAST/
Clustal X2	Larkin MA	http://www.ebi.ac.uk/tools/clustalw2
Gene Doc	Karl Nicholas	https://github.com/karlnicholas/GeneDoc
MEGA X	Sudhir Kumar	www.megasoftware.net
SnapGene	GSL Biotech	https://www.snapgene.cn/
Graphpad Pism9.5	GraphPad Software, Inc.	https://www.graphpad.com/

### Experimental model and study participant details

*C. plicata* with body length of 15 ± 5 cm and body weight of 220 ± 50 g were selected from Poyang Lake, Jiangxi Province, China, and were placed in an oxygenated room temperature aquarium (56 × 41 × 32 cm). Chlorella vulgaris was fed for one week.

### Method details

#### Total RNA extraction and cDNA synthesis

The total RNA was isolated from the examined tissues of *C.plicata* using Trizol reagent (TaKaRa, Japan) according to the manufacturer’s protocol. The extracted RNA was detected for concentration and purity. Select OD _600_ = 1.8-2.2 and qualify after agarose gel electrophoresis using HiScript II Q RT SuperMix for qPCR (gDNA wiper) kit for reverse transcription, and finally the product was stored at − 20°C.

#### Cloning the full length of *Cp*GSK-3β and *Cp*β-TrCP

*Cp*GSK-3β and *Cp*β-TrCP was searched from the transcriptome library that was provided by our laboratory, and was performed NCBI alignment analysis. The specific primers of GSK-3β-F3/R3, β-TrCP-F3/R3 were designed (Table S1). The open reading frames of *Cp*GSK-3β and *Cp*β-TrCP were cloned. According to SMARTer® RACE 5 ' / 3 ' Kit User Manual kit instructions (TaKaRa, Japan), the specific primers of GSK-3β-F1 / R1, GSK-3β-F2 / R2, β-TrCP-F1 / R1, β-TrCP-F2 / R2 (Table S1) were designed to obtain 3 ' and 5 ' RACE. After splicing by Snapgene, the full length of *Cp*GSK-3β and *Cp*β-TrCP was obtained.

#### Bioinformatics analysis

The amino acid sequence was translated using Expasy translate (https://web.expasy.org/translate/). The homology analysis was performed using BLAST (https://blast.ncbi.nlm.nih.gov/BLAST/). The amino acid sequence homology alignment was excuted using Clustalx and Gene Doc. The phylogenetic tree was constructed using MEGA software. All required sequences were obtained from the NCBI database (GenBank details are provided in Table S2).

#### Analysis expression of *Cp*GSK-3β and *Cp*β-TrCP in *C. plicata*

In order to study the expression of *Cp*GSK-3β and *Cp*β-TrCP in *C. plicata*, total RNA was extracted from eight different tissues of healthy *C.plicata*, including hepatopancreas, gill, kidney, mantle, foot, gonad, blood cells and adductor muscle. Specific primers were designed according to the cDNA sequences of *Cp*GSK-3β and *Cp*β-TrCP.The expression of genes in various tissues was detected by Real-Time PCR.

#### Peroxide and bacterial stimulation

Healthy molluscs were randomly divided into six groups, blank control group (healthy mussel without injection), PBS (control group), the experimental group LPS, PGN, Poly(I:C) (diluted with PBS) each injection of 100 μg. In addition, the last group was injected with 30% hydrogen peroxide, injected into adductor muscle of the molluscs. The tissues of hepatopancreas, gills and kidneys from the molluscs of each group were taken at 6, 12, 24, 48 and 72 h after injection.

The tissues of three molluscs were mixed into one sample, and three parallel samples were set up, which were placed in a non-RNase EP tube, were cooled in liquid nitrogen, and were stored at -80°C.

#### RNA interference

The specific primers with T7 promoter were designed according to *Cp*GSK-3β and *Cp*β-TrCP sequences. T7 RNAi Transcription Kit (Vazyme, Nanjing, China) was used to synthesize dsRNA. According to the experimental requirements, we selected 56 mussels of similar morphology and size, dividing them equally into seven groups of eight mussels each. The experiment included an LPS stimulation group and an LPS + dspEGFP-C1 group as the control. dsCpGSK-3β and dsCpβ-TrCP served as interference groups, with LPS + dsCpGSK-3β and LPS + dsCpβ-TrCP constituting the stimulated interference groups. The injection dose of LPS was 100 μg, while the injection doses of dspEGFP-C1, dsCpGSK-3β, and dsCpβ-TrCP were each 100 μg. The tissues of hepatopancreas, gill and kidney were collected at 12 and 24 h, respectively.

#### Real-time PCR analysis

SnapGene was used to design fluorescence quantitative specific primers and internal reference genes (Table S1). 6, 4, 2, and 0 M Urea was added for gradient dialysis that was changed at every 12 h, 4°C for 2 days. The primers for each target gene were detected by gradient and the product uniformity was analyzed by dissolution curve, and were screened according to the standard curve results, the template dilution multiple was determined. ChamQ Universal SYBR qPCR Master Mix was utilized on Bio-Rad CFX96. The reaction system was 2 × ChamQ Universal SYBR qPCR Master Mix 10 μL, RT-F 0.4 μL, RT-R 0.4 μL, cDNA 1 μL, ddH_2_O 8.2 μL. The reaction procedure was 95°C, 2 min, 95°C, 5s, 58°C, 30s, 72°C, 30 s, 40 cycles, 95°C, 5 s, 65°C. 5 s, 95°C, 5 min.

#### Subcellular localization

*Cp*GSK-3β and *Cp*β-TrCP were inserted into pEGFP-C1 plasmid (primer sequence is shown in Table S1). The HEK-293T cells were seeded in a 35 mm culture dish (DMEM + 10% FBS + 1% penicillium streptomycin) and were cultured in 37°C, 5% CO_2_ incubator for 12 h. The adherent and growth status of the HEK-293T cells were observed. If the cell aggregation rate was about 80%, the transfection could be carried out.

The transfection reagent was prepared according to the instructions of jetOPTIMUS® *in vitro* DNA transfection reagent, and the plasmid was transfected into the culture dish and was cultured for 48 h. The nucleus was stained with DAPI (Beyotime, Shanghai, China) staining solution. Finally, the distribution of green fluorescence was observed under a microscope.

#### Protein expression, purification and concentration determination

The final concentration of 0.5 mM β-D-thiogalactoside (IPTG) was added to pET-30a-CpGSK-3β bacterial solution and was induced at 37°C for 12 h. The final concentration of 2 mM IPTG was added to pET-28a-SUMO-Cpβ-TrCP bacterial solution and was induced at 37°C for 8 h. The induced bacterial solution was centrifuged at 12000 rpm for 10 min, the supernatant was discarded. After adding 40 mL PBS to re-suspend the bacteria on ice, the bacteria were ultrasonically broken and were centrifuged. The supernatant was discarded, which was washed twice with buffer A (5 mM EDTA, 50 mM Tris · HCL, PH = 8), was centrifuged at 12,000 rpm for 10 min at 4°C, and was washed twice with buffer B (5 mM EDTA, 50 mM Tris · HCL, 2 M Urea PH = 8).

The collected precipitate was dissolved in buffer C (10 mM DTT, 0.1 M Tris · HCL, 8 M Urea PH = 8) for denaturation at 1 h. The supernatant was collected by centrifugation, which was put into the dialysis bag and put it into the dialysis solution (0.1 M Tris HCL, 5 Mm L-cysteine, 5 mM EDTA, 10% glycerol, PH = 8). 6, 4, 2 and 0 M Urea was added for gradient dialysis, and was changed at every 12 h, 4°C for 2 days.

The AKTA protein purification instrument was used for purification and the Ni-IDA column containing His tag was connected to the system. The system was washed with ddH2O at a flow rate of 1 mL/min and washed with 1 × binding buffer (0.5 M NaCl, 20 mM Tris HCL, 5 mM imidazole, PH = 7.9) and elution buffer (0.5 M NaCl, 20 mM Tris · HCL, 1 M imidazole, PH = 7.9). The sample after sterilization was loaded at a flow rate of 0.2 mL/min. After the sample was loaded, the resin was circulated with 1 × binding buffer at 1 mL/min and the impurity protein was eluted with washing buffer (0.5 M NaCl, 20 mM Tris · HCL, 60 mM imidazole, PH = 7.9). After the UV value was stable, 50%-90% elution buffer was used for elution, and the flow rate was 1 mL/min (1 mL per tube). The collection was stopped after the UV value decreased steadily.

The collected protein was detected by 10% SDS-PAGE. The protein consistent with the target protein band was dialyzed with PBS for 3 times to remove imidazole. The concentration of protein was determined by the Coomassie brilliant blue method and was stored at -80°C.

#### Yeast two-hybrid experiment

*Cp*GSK-3β and *Cp*β-TrCP were constructed in pGBKT7 (BK) and CpNrf2 was done in pGADT7 (AD) by homologous cloning (Table S1). The binding of *Cp*Nrf2 to *Cp*GSK-3β and *Cp*β-TrCP was determined by yeast two-hybrid principle using two-deficient and four-deficient medium screening. Strains AD + BK, AD + BK-GSK-3β, AD + BK-β-TrCP, BK + AD-Nrf2 were screened out. The transformed and re-suspended bacteria were inoculated into (SD / -Leu / -Trp) and (SD / -Leu / -Trp / -His / -Ade) at 28.5°C for 3-4 days.

#### GST-pull down and Western blot

GST-tagged protein and *Cp*Nrf2-Neh6 were added to four centrifuge tubes containing GST-Beads (Sangon Biotech, Shanghai, China). 4°C chromatography cabinet overnight incubation. After cleaning the beads, His-*Cp*GSK-3β and His-*Cp*β-TrCP were added and were incubated at 4°C for 4 h. The incubation protein was washed which was added with 6 × Protein Loading Buffer, and was heated at 100°C for 10 min to denature. SDS-PAGE electrophoresis was performed.

The protein glue was transferred to PVDF membrane (millipore, USA) and was done at 4°C for 90 min. The membrane was taken out, which was cleaned with 1 × TBST three times, and was covered with 5% skimmed milk powder on a shaker at room temperature for 1 h. The membrane was cleaned by 1 × TBST for 10 min, and was washed three times. The PVDF membrane was incubated with primary antibody overnight at 4°C, which was washed three times with 1 × TBST, was incubated with Goat Anti-Mouse Antibody for 1h at room temperature, and was washed three times with 1 × TBST. Exposure development was performed using ECL chemiluminescence hypersensitivity developer.

#### *In vitro* phosphorylation

The purified pGEX-4T-1-CpNrf2-Neh6 (1.25 μg) and pET-30a-*Cp*SK-3β (2.5 μg) were added to 100 μL reaction solution (10 mM MgCl_2_, 10 mM ATP, 40 mM MOPS, 1 mM EDTA, PH = 7.0) at 30°C for 30 min. 6 × Protein Loading Buffer was added and was heated at 100°C for 10 min for 12% SDS-PAGE. The protein gel was stained using the Shenggong phosphorylated protein gel detection kit (Sangon Biotech, Shanghai, China).

#### Disk diffusion assay

According to the method of section protein expression, purification and concentration determination in this paper, 200 μL bacterial liquid was induced and was evenly applied to the solid medium containing Kana ^+^ at 37°C for 30 min. The different concentrations of H_2_O_2_ (0、50、100、200、400 mM) was lightly put on the medium for 6 mm, in the incubator at 37°C overnight. The cross method was utilized to measure the inhibition zone diameter.

#### Cell viability assay

HEK-293T cells in the logarithmic growth phase were made into cell suspension that were inoculated on 96-well plates, were placed in a 37°C, 5% CO_2_ incubator, and were cultured for 24 h. The pCMV-HA-CpGSK-3β and pCMV-HA-Cpβ-TrCP (Table S1) plasmids were transfected into cells according to the method in 2.10. After 24 hours of culture, 100 mM H_2_O_2_ was added and was incubated in a 5% CO_2_ incubator at 37°C for 6 h. Finally, the cell viability was measured by CCK-8 kit (Beyotime, China).

#### Statistical analysis

All data analysis and graphing were performed using GraphPad Prism 10.3.0 software. Each experiment was repeated three times, and data are expressed as mean ± standard deviation (SD). P-values were analyzed using t-tests. P-values < 0.05 were considered statistically significant. Significant differences between groups were denoted as ∗, #, or ▲ (P < 0.05) and ∗∗, ##, or ▲▲ (P < 0.01). Homogeneity of variance was assessed using Brown-Forsythe or Bartlett's tests prior to t-tests.
