# Supplementary material for: GSK-3β/β-TrCP regulates Nrf2-mediated oxidative stress response in Cristaria plicata
Source: iScience. 2025 Nov 11;29(3):113995. doi: 10.1016/j.isci.2025.113995 (PMC13080398; doi:10.1016/j.isci.2025.113995)
Supplement: Document S1. Figures S1–S3 and Tables S1 and S2 [file mmc1.pdf]

iScience, Volume *29*

Supplemental information

GSK-3β/β-TrCP regulates Nrf2-mediated oxidative stress response in *Cristaria plicata*

Yile Hu, Jinhua An, Han Qiu, Qinglian Wu, Jianqing Li, Gang Yang, Baoqing Hu, and Chungen Wen

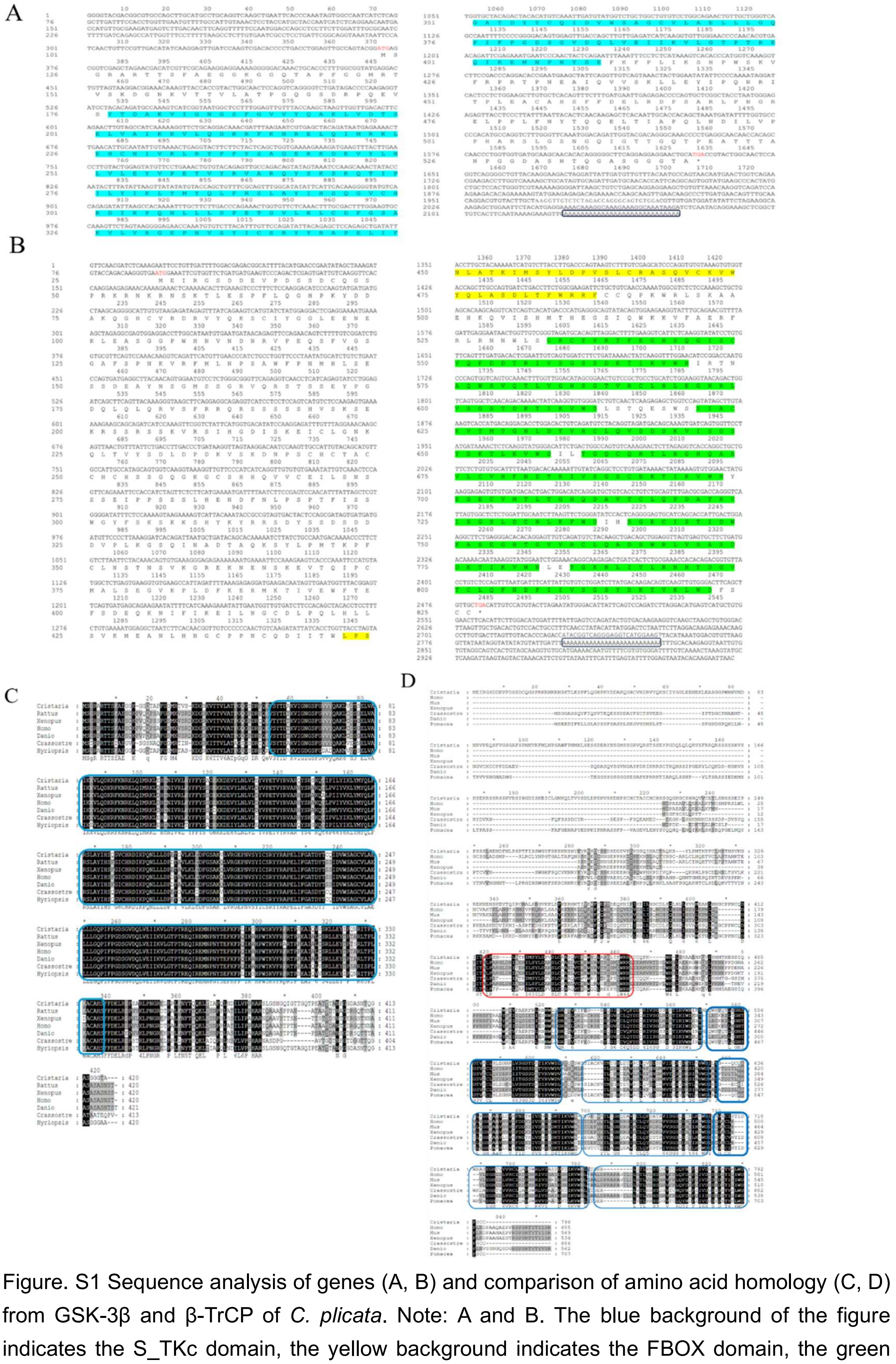

Figure. S1 Sequence analysis of genes (A, B) and comparison of amino acid homology (C, D) from GSK-3β and β-TrCP of *C. plicata*. Note: A and B. The blue background of the figure indicates the S_TKc domain, the yellow background indicates the FBOX domain, the green background indicates the seven WD40 domains, the blue box indicates the polyA tail, and the red font indicates the start codon and stop codon. C and D. orange box is S_TKc domain, red box is F-box domain, blue box is WD40 repeat motif.

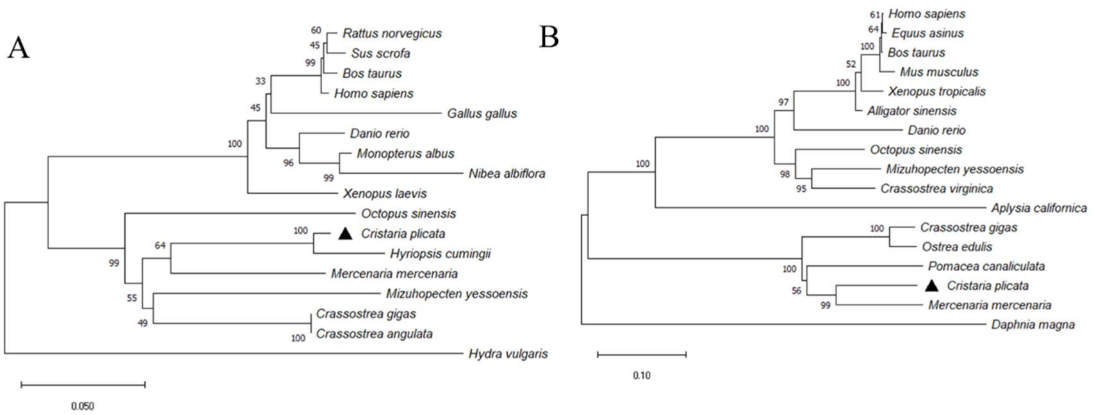

Figure S2. Phylogenetic tree of GSK-3β (A) and β-TrCP (B) of *C. plicata*. Note: The black triangle marked the species studied in this paper.

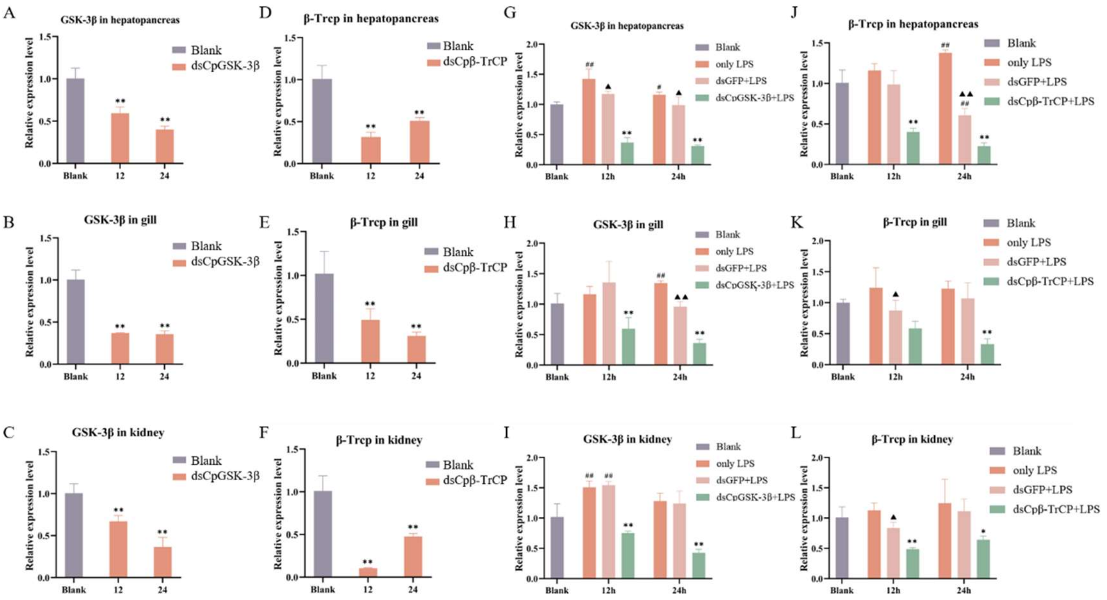

Figure S3. RNA interference efficiency. A–F show the detection of interference efficiency following RNA interference targeting GSK-3β and β-TrCP under non-stimulated conditions (labeled as dsCpGSK-3β and dsCpβ-TrCP, respectively). All data are presented as mean ± SD, n = 3. $P \leq 0.05$ is denoted by *, and $P \leq 0.01$ is denoted by **. G-L shows the detection of interference efficiency after RNAi targeting GFP, GSK-3β, and β-TrCP under LPS stimulation conditions (labeled as only LPS, dsGFP+LPS, dsCpGSK-3β+LPS, and dsCpβ-TrCP+LPS, respectively). Differences between groups and the blank group are indicated by #. Differences between LPS and LPS+dsGFP are marked with ▲. Significant differences between LPS+dsGSK-3β and the negative control LPS+dsGFP are denoted by *. Data are presented as mean ± SD, n = 3. $P \leq 0.05$ is indicated by a single #, ▲, or *; $P \leq 0.001$ is indicated by ##, ▲▲, or **.

Table S1 Primers sequences used in the research

Primer name	Primer sequence 5’ → 3’
Gene clone	
GSK-3β-F1	CCACTTCTCGTTGTCGAAGG
GSK-3β-R1	GCAGTCTGACCAGTCAGTTAC
β-TrCP-F1	TAGCTAAAGATGTACCAGAC
β-TrCP-R1	CCTTCTTGTCACAGTATCTG
GSK-3β-F2	CCGTACTGGCAACTCCAGGTCAGGGG
GSK-3β-R2	GCAGTTCCTCCTCCTGAAGCCCCCTG
GSK-3β-F3	GGTCTGCTGGCTGTGTCCTGGCAG
GSK-3β-R3	GACCTGGCATGTGGGGGCACC
β-TrCP-F2	CAACGATCTCAAAGAATTCCTGTTG
β-TrCP-R2	CCACAGCATGACTCATGTCCT
β-TrCP-F3	GCTAGAGGCGAGTGGAGGAC
β-TrCP-R3	GCTGAAGTCCCACAACTTGACTGTC
RT-PCR	
GSK-3β-qF	CCCCAGTGCTCGGCTACCTAATGGG
GSK-3β-qR	GGCTGTGGTTGTTGCCTCAGGGG
β-TrCP-qF	CCACTCAGGGGAGTGCATCAGC
β-TrCP-qR	CCGTCTGTGTGGTTTCGCAGAGTG
NQO1-qF	CGAGTGCGGACTTGTTGA
NQO1-qR	TCGCTTTCTTGGCTGTAA
Nrf2-qF	AATGCTACCCTCCCTACTCC
Nrf2-qR	GTGTTGTTGAAGCCAGTGAA
RT-actin-F	AAGGTTACGCCCTTCCTCAT
RT-actin-R	GCCATTTCCTGCTCAAAGTC
RNAi	
GSK-3β-Ri-TF	GCACCCTTTGGCGGTATG
GSK-3β-Ri-R	GGCACTTCCAAAGTCGCAAAG

GSK-3β-Ri-F	GCACCCTTTGGCGGTATG
GSK-3β-Ri-TR	GGCACTTCCAAAGTCGCAAAG
β-TrCP-Ri-TF	CCCAGACTCGAGTGATTGTC
β-TrCP-Ri-R	CCTTATCAGGGTCAAGGTCAG
β-TrCP-Ri-F	CCCAGACTCGAGTGATTGTC
β-TrCP-Ri-TR	CCTTATCAGGGTCAAGGTCAG
Prokaryotic expression	
30-GSK-3β-F	tggtggtggtggtgctcgagTGCAGTTCCTCCTCCTGAAG
30-GSK-3β-R	ccatggctgatatcggatccATGAGCGGTCGAGCTAGAAC
28a-SUMO-TF	GAGAACAGATTGGTGGATCCCTTCCACAGCTACACCTCCTTT
28a-SUMO-TR	TGGTGGTGGTGGTGCTCGAGGCAACAGCTGAAGTCCCACAA
4T-1-NF	CGCGTGGATCCCCGGAATTCTCTGGCAGCATCAACCAGAC
4T-1-NR	GTCAGTCACGATGCGGCCGCTCAATATGGTTTGTATCTGCGGG
Subcellular localization	
GFP-GSK-3β-F	GTCCGGACTCAGATCTCGAGCTATGAGCGGTCGAGCTAGAAC
GFP-GSK-3β-R	TATCTAGATCCGGTGGATCCTGCAGTTCCTCCTCCTGAAG
GFP-β-TrCP-F	TCCGGACTCAGATCTCGAGCTATGGAAATTCGTGGTTCTGATGA
GFP-β-TrCP-R	TATCTAGATCCGGTGGATCCGCAACAGCTGAAGTCCCACAA
GFP-Nrf2-F	GTCCGGACTCAGATCTCGAGCTTCTGGCAGCATCAACCAGAC
GFP-Nrf2-R	TATCTAGATCCGGTGGATCCAATATGGTTTGTATCTGCGGGACT
Yeast two-hybrid	
BK-GF	tggccatggaggccgaattcATGAGCGGTCGAGCTAGAAC
BK-GR	ttatgctagttatgcggccgcTGCAGTTCCTCCTCCTGAAG
BK-TF	tggccatggaggccgaattcCTTCCACAGCTACACCTCCTTT
BK-TR	ttatgctagttatgcggccgcGCAACAGCTGAAGTCCCACAA
AD-NF	CCATGGAGGCCAGTGAATTCTCTGGCAGCATCAACCAGAC
AD-NR	CGATTCATCTGCAGCTCGAGCAATATGGTTTGTATCTGCGGGACT
Eukaryotic expression	
HA- GSK-3β-F	GGCCATGGAGGCCCGAATTCGGATGAGCGGTCGAGCTAGAAC

HA- GSK-3β-R	TGTCTGGATCCCCGCGGCCGCTGCAGTTCCTCCTCCTGAAG
HA-β-TrCP-F	GGCCATGGAGGCCCGAATTCGGATGGAAATTCGTGGTTCTGATG
HA-β-TrCP-R	TGTCTGGATCCCCGCGGCCGCGCAACAGCTGAAGTCCCACAA

Table S2 Species name and accession number used in phylogenetic tree

Gene	Species	GenBank
GSK-3β	*Rattus norvegicus*	NP_114469.1
	Xenopus laevis	NP_001083752.1
	Bos taurus	NP_001094780.1
	Homo sapiens	NP_002084.2
	Sus scrofa	NP_001121915.1
	Danio rerio	NP_571456.1
	Hydra vulgaris	AEM76871.1
	Crassostrea gigas	XP_011452760.1
	Crassostrea angulata	CCN27373.1
	Mizuhopecten yessoensis	OWF50471.1
	Monopterus albus	XP_020465114.1
	Octopus sinensis	XP_029636743.1
	Gallus gallus	XP_040516343.1
	Nibea albiflora	KAG8000603.1
	Hyriopsis cumingii	UYI35619.1
	Mercenaria mercenaria	XP_045207912.1
β-TrCP	*Daphnia magna*	JAM74715.1
	Homo sapiens	KAI4077194.1
	Mus musculus	AAD41025.1
	Xenopus tropicalis	NP_001016386.1
	Bos taurus	DAA14816.1
	Equus asinus	XP_014693125.1
	Octopus sinensis	XP_029644679.1
	Pomacea canaliculata	XP_025093821.1
	Mizuhopecten yessoensis	OWF43849.1
	Crassostrea gigas	XP_011415998.2
	Crassostrea virginica	XP_022341985.1
	Aplysia californica	XP_012941965.2
	Alligator sinensis	XP_025051050.1
	Danio rerio	XP_005173126.1
	Mercenaria mercenaria	XP_045164454.1
	Ostrea edulis	XP_048740230.1
